# Demographic and clinical characteristics of our patients diagnosed with laryngeal dystonia

**DOI:** 10.1007/s00405-024-08688-9

**Published:** 2024-05-07

**Authors:** Orhan Asya, Ömer Tarık Kavak, Hatice Ömercikoğlu Özden, Dilek Günal, Necati Enver

**Affiliations:** 1 Department of Otorhinolaryngology, Pendik Training and Research Hospital, Marmara University Faculty of Medicine, Fevzi Çakmak, Muhsin Yazıcıoğlu Street, 34899 Istanbul, Turkey; 2 Department of Neurology, Pendik Training and Research Hospital, Marmara University Faculty of Medicine, Fevzi Çakmak, Muhsin Yazıcıoğlu Street, 34899 Istanbul, Turkey

**Keywords:** Laryngeal dystonia, Spasmodic dysphonia, Voice, Botulinum toxin

## Abstract

**Purpose:**

Laryngeal dystonia (LD) is a focal dystonia affecting laryngeal musculature with no known etiology or cure. The present study evaluated the sociodemographic and clinical features of patients diagnosed with LD.

**Materials and Methods:**

All patients diagnosed with LD at our University Hospital’s Ear, Nose, and Throat Department between January 2017 and July 2023 were retrospectively analyzed. The study included 43 patients.

**Results:**

Out of the 43 patients, 19 (44%) were male. At the time of diagnosis, the mean age of the patients was 35.1 years (ranging from 17 to 65 years). The mean elapsed time between the first symptom onset and the first diagnosis was 49.2 months (min. 4 months, max. 240 months). Of the participants, 94% had adductor-type LD. None of the patients had a family history of LD. Of the patients, 9 (20%) experienced a life-altering event or trauma just before the onset of symptoms. All patients who consumed alcohol reported symptom relief with alcohol intake. A total of 67.6% of patients stated that their symptoms were triggered by stress. All of our patients received at least one Botulinum toxin injection, with an average of 2.75 dosages per patient.

**Conclusion:**

The gender distribution was approximately equitable between males and females. There was a tendency for men to receive a diagnosis earlier than women following the manifestation of symptoms. A significant number of patients associate the emergence of their symptoms with a stressful event or traumatic experience. This study represents the initial investigation into the sociodemographic characteristics of patients within the Turkish population.

## Introductıon

Laryngeal dystonia (LD) is a focal neurological dystonia characterized by intermittent and involuntary spasms caused by activation of the intrinsic laryngeal muscles during phonation, causing task-specific vocal fluency impairment [1]. LD is an uncommon form of dysphonia that does not include a phonatory organ disease or paralysis [2, 3]. Vocational incapacity and social isolation are the two most common distressing conditions caused by LD in patients [4, 5]. The pathophysiology of LD is inadequately comprehended, and the process of diagnosis presents challenges [6].

LD is characterized by its multifactorial etiology, and genetic variants are considered important risk factors for the development of this disorder. Nevertheless, traditional linkage studies in LD have been severely limited by low penetrance of dystonia, rare availability of large families, phenotypic discordance between affected family members, and late age at onset. While 12% of LD patients have a family history of dystonia [7], a specific gene for LD has not been identified. Among other forms of dystonia-causing gene mutations that have been identified, laryngeal involvement has been reported in patients with generalized and segmental dystonia who are carriers of the DYT1, DYT4, DYT6, DYT25, and DYT28 mutations [8]. Only 1 case of focal adductor LD (AdLD) with DYT25 (GNAL) mutation and without any other co-occurring forms of dystonia, a family history of dystonia, or other movement disorders has been reported to date [9]. Not only are the mutations in specific genes linked with dystonia but polymorphisms also play a role. While mutations of the TOR1A gene are responsible for early onset segmental dystonia that rarely involves laryngeal dystonia, polymorphisms in the same gene have been associated with adult-onset, primarily focal dystonia, including LD and even a decreased risk of developing dystonia.

Imaging studies have played a crucial role in investigating the specific pathophysiology of LD. In the study conducted by Walter et al., transcranial sonography revealed hyperechogenicity in the lenticular nucleus and caudate nucleus in 12 out of 14 patients with AdLD [10]. Subsequent neuroimaging studies have demonstrated that the pathophysiology of LD encompasses extensive alterations in both the function and structure of brain networks. These alterations affect not only the basal ganglia, but also higher-order motor and associative cortical regions, the thalamus, and the cerebellum [11]. Distinct symptoms in LD may be linked to disorder-specific network alterations [12]. Impaired brain plasticity and neurotransmission in LD also point to other mechanisms in dystonia pathophysiology, including abnormal dopaminergic and GABAergic function and maladaptive plasticity [13]. From a clinical point of view, the significance of variations in these regions is apparent from their diagnostic potential in the successful machine-learning classification of LD, achieving up to 98.8% accuracy in objectively diagnosing this disorder [14].

Despite the LD studies conducted in recent years, epidemiological data are insufficient due to the lack of studies observing the LD patient group in the long term. Although the diagnosis of LD can be challenging, the patient’s history, laryngoscopic examination, and phonatory characteristics of the voice often make the diagnosis. In a study by Hyodo et al. in Japan [15], the prevalence was 3.5–7.0/100,000, similar to that in Rochester (NY, USA) and Iceland[16]. Women were four times more likely to be affected than men. The mean age of onset varies between 40 and 50 years. Although rare, familial relationships have been shown in a few studies [17].

There are three forms of LD: adductor, abductor, and mixed. Adductor phenotype is the most common type (90–95%), in which intermittent glottal closure blocks expiratory airflow and causes a strained and hoarse voice quality with vocal interruptions. In abductor LD (AbLD), spasms of the intrinsic laryngeal abductor muscle open the vocal folds during speech, resulting in an intermittent breathy voice or aphonia [2–4]. Mixed LD exhibits both vocal symptoms and is rarely seen.

Regardless of the subtype of LD, patients have difficulty speaking naturally and effectively, which has a significant impact on their professional and social communication [18, 19]. Some tactile or proprioceptive exercises, such as chewing or head bracing, can enhance speech for certain people [20]. The mechanism behind this behavior is unknown. Singing or laughing can boost speech fluency, possibly capitalizing on the task-specific character of dystonia [21].

Botulinum toxin (BT) injection into the laryngeal musculature is the most common and effective treatment method for LD. Surgery and voice therapy are other treatment options. BT inhibits acetylcholine release at the neuromuscular junction by reducing muscle activity. The target muscles are thyroarytenoid in AdLD patients and posterior cricoarytenoid in AbLD patients. Surgical interventions include type 2 thyroplasty [22, 23], thyroarytenoid myectomy [24, 25], and selective laryngeal denervation-reinnervation [26]. Voice therapy also aims to reduce strong glottal closure, but its effectiveness is limited.

There is currently no research on the prevalence of LD in Turkey. The objective of our study was to identify the demographic and clinical features of individuals with LD identified at our clinic which is among the few tertiary centers in Turkey that specialize in LD follow-up. Further research is necessary on LD prevalence, likely etiologies, treatment modalities, and diagnosis.

## Materials and methods

The sample population of the study consisted of patients diagnosed with LD who were followed at the Ear, Nose, and Throat Department of a tertiary medical center. The study was approved by a tertiary medical faculty Ethics Committee for Clinical Research on 27/10/2023 with number 09.2022.1449. Because the study was designed retrospectively, informed consent was not obtained from the patients. Descriptive statistic was used to present data. The study included patients who were diagnosed following admission and subsequently received treatment at our clinic. The diagnosis was made by a speech therapist and laryngologist with at least ten years of experience, employing a three-tiered technique as proposed by Ludlow et al. [27]. Firstly, screening questions were utilized to indicate potential LD. Secondly, a comprehensive speech examination was conducted to further assess and identify likely LD. Finally, the laryngoscopic examination was made for a definitive diagnosis. Neurologists also assessed the patients for potential coexisting movement disorder. A retrospective investigation was conducted on a cohort of 43 patients diagnosed with LD between January 2017 and July 2023. All patient data were obtained from past medical records and missing data was fulfilled with telesurveys. Sociodemographic characteristics of the patients, the age of symptom onset, and the time elapsed between the first onset of symptoms and the diagnosis were noted. Family history of dystonia-like symptoms and the existence of factors that trigger or alleviate symptoms were questioned. BT injections are the treatment of choice and are performed under local anesthesia with the help of laryngeal EMG. Each patient was questioned regarding the frequency of injections, the onset and peak days of injection effectiveness, and the length of injection effectiveness. Additionally, the dosage of injections was obtained from medical records.

## Results

The study included 43 patients and Table 1 summarizes the results. 19 (44%) were male and 24 (56%) were female. The patients' mean age at the time of diagnosis was 35.1 years (17–65 years). The mean age at the onset of the first symptoms in patients before diagnosis was 31.2 years (16–53 years). The average time from the first date of symptoms to the diagnosis of LD in our patients was 49.2 months (4–240 months), while this period was 42 months (4–120 months) for men and 64 months (6–240 months) for women. Thus, there was an approximately four-year delay from symptom onset to diagnosis in our study population. In our study, 40 participants (94%) had AdLD, and three (6%) had AbLD. Of the patients, nine (20%) reported experiencing a major stress or trauma that could affect their lives just before symptom onset. All patients were admitted to a hospital more than once until the diagnosis was made.

**Table 1:** Demographics and clinical characteristics of the patients

Characteristic	Patients with laryngeal dystonia (N = 43)
F/M	1.26/1
Mean age at the time of diagnosis	35.1 years (17–65 years)
Mean age at the onset of the first symptoms in patients prior to diagnosis	31.2 (16–53 years)
The average time from the first date of symptoms to the diagnosis	49.2 months (4–240 months)
N AdLD/N AbLD	40/3
Concomitant vocal tremor	2 (7%)
Alcohol consumption	10 (23%)

None of our patients had a family history of LD. Previous research has indicated a correlation between LD and an increased incidence of movement problems. With respect to movement disorders, 3 of our patients have co-existing vocal tremor. 2 out of the 43 patients had additional neurological disease, specifically peripheral nerve entrapments. In our study, 10 (%23) patients consumed alcohol daily basis and all patients who consumed alcohol claimed that their symptoms improved after alcohol consumption. Conversely, 24 (55%) patients reported worsening of their symptoms under emotional stress or anxiety. Our patients with AdLD undergo bilateral thyroarytenoid muscle (TA) botulinum toxin injections using a hollow-bore 27 gauge Teflon-coated EMG needle with EMG control. In the AdLD group, patients who did not achieve the intended outcome following the injection while being monitored by EMG, or in cases where EMG was not accessible, were given TA botulinum toxin injections employing flexible laryngoscopy in an office setting. Before administering the injections, patients received 5 cc of 2% lidocaine through the cricothyroid membrane to generate local anesthesia for the TA botulinum toxin injection process. On average, they receive 2.7 injections, with a minimum of 1 injection and a maximum of 20 injections. All of our data is based on injections of Botox^®^ (Allergan, Inc., Irvine, CA, USA). Our average dose is 1.5 U/0.1 ml per vocal fold. The findings for the entire adductor group indicate that the average time for the positive effects to start is 2.7 days. The average maximum effect occurs after 7 days, and the average length of improvement is 12 weeks. In the AbLD, we treated 3 patients with an average dose range of 2.5 U/0.1 ml units. given separately and spaced 2 weeks apart. The strategy for the AbLD group is to treat via posterior cricoarytenoid muscle (PCA) injections. They all received 1 injection for each PCA. In all three AbLD cases, the breathy voice persisted to some extent, despite a satisfactory reduction following the administration of the injection. The objective is to modify the dosage in subsequent injections.

## Discussion

### Epidemiological studies

In Turkey, there is no comprehensive study to determine the prevalence of LD; hence, additional research is required at the national level. Due to the unavailability of medical records for all individuals residing in the district where the center is located, and the fact that patients from various provinces of Turkey are being followed in our center, it is not feasible to determine the prevalence in our study. Several epidemiological investigations of LD have been published worldwide. Through conducting research on patients diagnosed with dystonia Duffey et al. (1998) and the Epidemiological Study of Dystonia in Europe Collaborative Group reported lower prevalence rates of 0.8/100,000 in Northern England and 0.7/100,000 in eight European nations, respectively [28, 29]. In 2002 Konkiewitz et al. [30] surveyed patients with dystonia and discovered an LD incidence of 1/100,000 in Munich, Germany (0.4–1.5). Pekmezovic et al. [31] collected patient data on idiopathic focal dystonia from both hospital inpatient and outpatient case records in Belgrade, Yugoslavia, encompassing all neurological facilities in the region. They found a prevalence of 1.1/100,000 for LD. Using a survey of social health services, Asgeirsson et al. [32] reported that the prevalence of LD in Iceland was 5.9/100,000. This was the first countrywide LD study in the world, made possible by the country’s very small population (approximately 288,000). In 2020, Hyodo et al. did a comprehensive search in The Japan Medical Abstract Society database, PubMed, and Google Scholar database up until the year 2019, speculated that the LD prevalence in Japan was 3.5–7.0/100,000 [15]. Nevertheless, most of the data collected in epidemiological research are derived from dysphonic patients who had treatment. Consequently, incorporating untreated patients can yield more precise outcomes. However, the absence of a universally recognized diagnostic approach for the LD and the limited availability of expert facilities in each sub-region of the countries contribute to the delayed nature of the diagnosis process and may exclude untreated LD patients for epidemiological research.

Epidemiological research has demonstrated that the prevalence of LD in Japan is greater than in Munich and European countries, and comparable to the rates observed in Rochester and Iceland. It has been reported that individual socioeconomic status is closely related to access to healthcare services [33]. Nevertheless, the presence of universal healthcare coverage as a global policy in Japan and European countries [34, 35], coupled with their high socioeconomic status, implies that they likely face comparable challenges when it comes to accessing the healthcare system. The differences may arise from variations in the research methods employed and the level of awareness among physicians and patients regarding LD. However, further studies are needed to investigate these hypotheses and it is imperative to conduct national-level studies as reports from specific geographical regions may not accurately represent the overall population.

### Female‒male predominance

LD is more common in women than in men. More than 65% of the affected population is women and the average age of symptom onset is 45 years [36]. Although most studies in the literature show female predominance, Nerurkar et al. [37] reported an 80% male predominance in their study. Consistent with the literature, we revealed a female predominance. However, the interpretation of the predominance of the disease between males and females is necessarily constrained in our study due to the small sample size.

### Psychological/traumatic event prior to first symptoms or stress as a triggering factor

In this study, "life-altering event prior to symptoms" was defined as the actual patient-observed situation during which symptoms of LD first appeared or became apparent to the patient. Stress was also questioned as a triggering factor. 9 (20%) patients associate their onset with a particular event, while others do not. Moreover, stress was identified as the predominant trigger among the majority of patients (26 out of 43, 60%), consistent with previous findings reported in the existing literature [38, 39].

### AdLD-AbLD

AdLD accounts for the majority of LD. In our research, 40 (94%) of the participants had AdLD. In the literature, the prevalence of AdLD ranged from 82 to 96%. Blizter [7] and Tisch et al. [40] reported this rate to be 82% and 89.4%, respectively. Tanner et al. [41] demonstrated a greater proportion of overall AdLD patients with 96.6%. AbLD prevalence ranged from 1.3 to 17% in previous studies [7, 40–42], while in our study, there were three (7%) affected individuals.

### Age of onset

In the literature, the average age of onset has been reported to be between 45 and 56 years [30–32, 40, 41]. In Japan, Hyodo et al. discovered that 60% of patients were in their twenties or thirties and that the mean age of onset was 31 years [15]. In our study, the mean age at the time of diagnosis was 35.1 years (17–65 years).

### Time of diagnosis onset

In the present study, the mean age at which individuals experienced their first symptoms before being diagnosed was 31.2 years which was slightly younger than what most previous studies report [27, 43]. Many physicians and patients may be unfamiliar with the clinical features of LD leading to delayed diagnosis [15, 43]. Hyodo et al. [15] reported that the duration between the onset of symptoms and the subsequent diagnosis exceeded 10 years for 20% of the subjects in their study. Similarly, Creighton et al. [43] conducted research that revealed an average duration of 4.5 years between symptom onset and diagnosis. In our study a delay in diagnosis was observed, consistent with previous findings reported in the literature. The average time from the first date of symptoms to the diagnosis of LD in our patients was 49.2 months, while this period was 42 months for men and 64 months for women. This duration is not only too long for patients who struggle with the social and professional challenges of LD symptoms but also wastes healthcare resources from a broader public health viewpoint.

### Genetic factors

LD is characterized by its multifactorial etiology, and genetic variants are considered important risk factors for the development of this disorder. Additionally in study conducted by Guiry et.al, 25.3% of their LD patients reportedly have a family history of dystonia [44]. LD genetics presents unprecedented challenges for the discovery of a causative mutation. Multi-institutional studies are needed to overcome challenges associated with the sample size required for conducting large-scale genomic studies in LD. At present a single case of isolated focal LD with DYT25 (GNAL) mutation has been identified [9], and the polygenic risk of dystonia, including LD and involving genes implicated in synaptic transmission and neural development, has been determined [45]. Although evidence of a clear link between specific genes and larynx-involving dystonia has been shown, the diagnostic and prognostic utility of genetic screening in clinical settings is still not clinically impactful [36]. These genotype-specific changes, however, can provide an important step toward the future description of imaging markers and potential targets for new LD diagnostics and therapeutic interventions. In our study, none of the patients had a family history of dystonia, and genetic analysis was not conducted on any of the patients.

### Comorbid movement disorders

Patients with LD are at approximately a 7% risk of spread of dystonia to another body part [46]. Additionally White et al. [42] demonstrated that patients with LD had significantly higher rates of comorbid tremor than controls when combining vocal and nonvocal tremor. They also reported that the prevalence of comorbid vocal tremor was significantly higher in patients with LD. In our study, 3 of our patients have coexisting vocal tremor. It has been reported that up to 11.8% of LD patients have a family history of other movement disorders [44]. Unlike the previous research [44], our group of patients with LD did not report a family history of movement disorders.

### Extrinsic risk factors

Although there is no direct evidence to suggest that LD is caused primarily by an extrinsic factor, case–control studies indicate a notably increased occurrence of certain health and environmental events such as upper respiratory tract infection, mumps and excessive voice usage in LD patients compared to the general population. Tanner et al. [41] noted that having a history of mumps or measles increases the likelihood of LD. Our study identified only one patient with a history of mumps. Mumps can be encountered as an infection in a significant portion of the normal healthy population in our society. Therefore, the fact that only one patient was identified with mumps in our study cannot be interpreted as mumps as a predisposing factor for LD. Recent research further also showed that stressors altering sensory feedback from the larynx such as recurrent upper respiratory tract infection may represent an extrinsic risk for LD and contribute to altered sensorimotor preparation and integration in susceptible individuals [47]. In our study one patient had a history of upper respiratory tract illness prior to the onset of LD symptoms. As none these extrinsic factors are rare events, a complex interactive process may contribute to pathogenesis in a small proportion of those at risk.

### Relieving, worsening factors

Kirke et al. [48] observed that alcohol consumption improves voice complaints in up to 58% of LD and LD/vocal tremor patients, with possible effects lasting between three and four hours. Although a considerable number of patients reported using alcohol in professional and social settings to improve their voice quality, this form of self-management of symptoms may be detrimental in the long term. In our study, 23% of the patients used alcohol daily, and all of the patients who used alcohol stated that their complaints regressed after consuming alcohol. As in other studies, most patients reported worsening symptoms when stressed or angry.

### Treatment

The standard therapy for LD is symptomatic control with BT chemodenervation [49]. This is backed by a substantial amount of research attesting to its efficacy in a variety of investigations, particularly for the simple adductor variant of the disease. The efficacy of voice treatment as a standalone intervention is limited, but it may have potential benefits when used in conjunction with behavioral therapy [50]. This combined approach could potentially target the compensatory behaviors that are observed alongside LD. Surgical therapy efforts precede the invention of BT by a decade, but their long-term effectiveness has not been demonstrated; therefore, further studies are needed. In individuals with abductor LD and dystonia with tremor, the symptom improvement with surgery or BT remained unsatisfactory. In our study, all patients received at least one BT injection transcutaneously into the TA muscle for the AdLD group and the PCA muscle for the AbLD group. These injections were administered through the cricothyroid membrane under electromyography guidance. The injection was found to have a positive impact on the self-evaluation of all patients. None of the patients showed a preference for surgical treatment. Speech therapy was employed solely for the aim of distinguishing between LD and Muscle tension dysphonia (MTD) in cases where there was clinical suspicion.

## Conclusion

This study analyzed the age, sex distribution, and clinical features of 43 patients diagnosed with LD at a single center in Turkey. The scarcity of research on the topic has resulted in a lack of relevant statistics regarding the prevalence of LD in Turkey. Therefore, it is necessary to conduct multicenter studies to establish the prevalence in Turkey. This study represents the largest cohort of Turkish patients diagnosed with LD at a single hospital thus far.

Although female predominance has been reported for the LD patient group in the literature, our study observed an almost equal distribution (M/F, 19/24). Studies have shown that movement disorders are associated with a higher rate in patients with LD. In our investigation, we detected concomitant vocal tremor in three cases, while the remaining individuals had isolated LD. While there has been a familial relationship in a small part of the studies on LD performed thus far, our study found no familial history in the LD patient population.

There is currently no standard diagnostic method for LD. The time elapsed between the onset of symptoms and diagnosis can take years. During this time, hospital admissions are seen more than once. Treatment is symptomatic therapy through BT chemodenervation. This is supported by a large body of literature confirming the efficacy of BT in many different studies, particularly in the uncomplicated adductor form of the disorder. Research into surgical treatment predates the development of BT therapy over a decade. However, its long-term effectiveness has not been proven, which points to the need for further research.

## Data Availability

The data that support the findings of this study are available from the corresponding author upon reasonable request.
